# Supplementary material for: Coherent tunnelling across a quantum point contact in the quantum Hall regime
Source: Sci Rep. 2013 Mar 11;3:1416. doi: 10.1038/srep01416 (PMC3593222; doi:10.1038/srep01416)
Supplement: Supplementary Information — for [file srep01416-s1.pdf]

Supplementary Information for
"Coherent tunnelling across a quantum point contact in the quantum Hall regime"

F. Martins[1], S. Faniel[2], B. Rosenow[3], H. Sellier[4], S. Huant[4], M. G. Pala[5], L. Desplanque[6], X. Wallart[6], V. Bayot[1,4] and B. Hackens[1]

[1] *IMCN/NAPS, Université catholique de Louvain, Louvain-la-Neuve B-1348, Belgium*

[2] *ICTEAM/ELEN, Université catholique de Louvain, Louvain-la-Neuve B-1348, Belgium*

[3] *Institute for Theoretical Physics, Leipzig University, Leipzig D-04009, Germany*

[4] *Institut Néel, CNRS and Université Joseph Fourier, Grenoble F-38042, France*

[5] *IMEP-LAHC, Grenoble INP, Minatec, Grenoble F-38016, France*

[6] *IEMN, Cité scientifique, Villeneuve d'Ascq F-59652, France*

(Dated: December 17, 2012)

PACS numbers: 73.21.La,73.23.Ad,03.65.Yz,85.35.Ds

ORIGIN OF THE BACKGROUND IN SGM

Fig. S1 allows inferring the origin of the broad background in SGM images. By sweeping $V_{\rm tip}$ below 0 V, the SGM maps measured at 100 mK shown in Figs. S1(a-c) reveal concentric fringes marking the presence of a QHI near the saddle point of the QPC. The diameter of circling fringes increases with decreasing $V_{\rm tip}$, consistent with the observations in Fig. S 1. Importantly, the SGM pattern around the QPC exhibits a strong variation that adds to the concentric fringes on Figs. S1(b-c). The origin of the stronger contrast is found by positioning the tip near the saddle point of the QPC and continuously decreasing $V_{\rm tip}$ (Fig. S1(d)). The main trend shows step-like increases of the device resistance which can be understood by invoking ES reflections at the constriction. Decreasing $V_{\rm tip}$ raises the energy of the saddle point and decreases the local filling factor ν^* near the constriction. Every time ν^* passes a half integer value, an ES is totally reflected and the device resistance shifts to the next plateau given by $R = h/e^2(1/N^* - 1/N)$ [1, 2] (brown dashed lines in Fig. S1(d)), where N^* is the (integer) number of transmitted ES at the constriction. The presence of oscillations superimposed on the first plateau at $N^* = 5$, similar to those around $V_{\rm tip} = 0$ V, indicate that the QHI is active even when one ES is reflected.

CALCULATION OF TRANSMISSION COEFFICIENTS ACROSS A QHI

Here we summarize the details concerning the determination of the coefficients of transmission across the tunnel barriers defining the QHI in the coherent regime (we assume here that all barriers have equal transmission coefficients). In this work it was found that the peak-to-peak amplitudes in the coherent regime (ΔR) were within the following intervals: 170Ω ($N^* = 5$) $< \Delta R <$ 200Ω ($N^* = 6$). Assuming that $\Delta R = h/e^2(1/(N^* - T_{\rm t}) - 1/N^*)$ [1, 2] where $T_{\rm t}$ is the total transmission through the QHI (i.e. taking into account the two barriers defining the QHI), we conclude that $T_{\rm t}$ is the interval: $0.16 < T_{\rm t} < 0.27$.

In Fig. S 3 we draw the two models considered in the main article. In the following subsections we deduce the coefficients of transmission of the tunnel barriers for the two different situations presented in Fig. S 3(a) and (b).

QHI at the centre of a QPC

We first consider the situation represented in Fig. S 3(a) where a QHI is located at the centre of a QPC. In this case we assume that coherence is maintained during the multiple reflections. For an off-resonance condition and assuming $T_1 = T_2 = T_c$, T_c is given by: $T_c = 2T_t(1 + T_t)$ [4], which implies that $0.27 < T_c < 0.42$.

Interferometer formed around the QPC saddle point

In the case of an interferometer formed around the saddle point of a QPC, as illustrated in Fig. S 3(b), we compute the reflection coefficient $1 - T_t$, taking into account interferences between different semiclassical paths for electrons : 1) a direct path along the edge state, which does not include transmission through the tunnel barriers, and 2) paths including multiple transmissions through the tunnel barriers. The reflection coefficient is then given by [4]:

$$1 - T_t = \frac{(1 - T_3)(1 - T_4)}{1 + T_3 T_4 - 2\sqrt{T_3 T_4} cos(\varpi)} \quad (1)$$

where ϖ is the phase difference accumulated along the two types of trajectories and T_3 and T_4 are the transmissions at each side of the saddle point. Assuming that $T_3 = T_4 = T_{3,4}$, we obtain: $0.043 < T_{3,4} < 0.078$.

---

[1] Aoki, N., da Cunha, C. R., Akis, R., Ferry, D. K. & Ochiai, Y. Imaging of integer quantum Hall edge state in a quantum point contact via scanning gate microscopy. Phys. Rev. B 72, 155327 (2005).

[2] Büttiker, M. Absence of backscattering in the quantum Hall effect in multiprobe conductors. *Phys. Rev. B* **38**, 9375-9389 (1988).

[3] Martins, F. *et al. submitted.*

[4] Datta, S. Electronic Transport in Mesoscopic Systems (Cambridge Univ. Press, Cambridge, 1995).

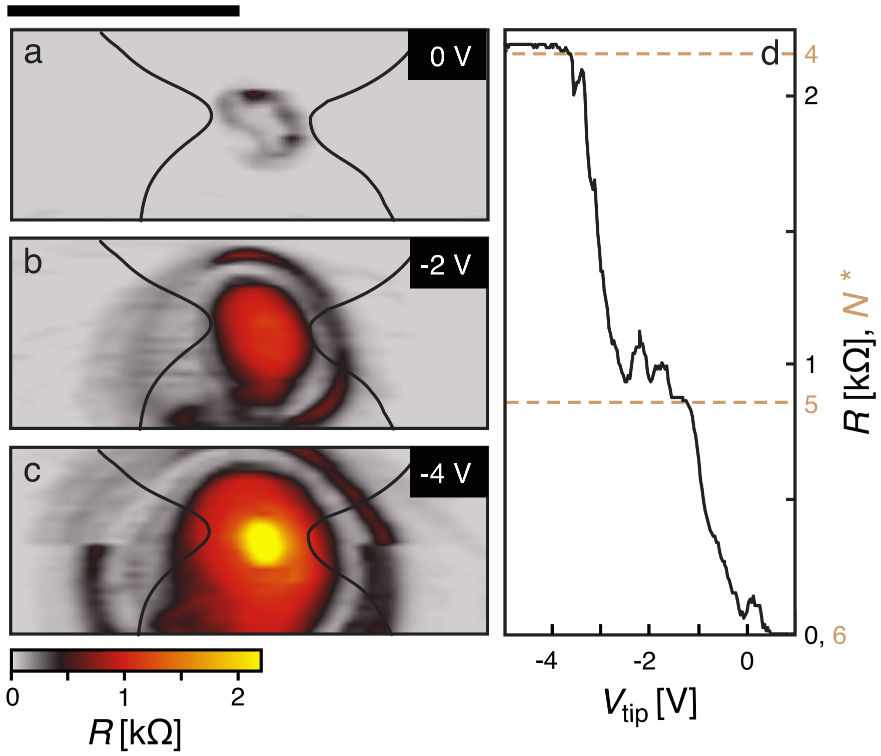

FIG. S 1: (a-c) Consecutive SGM images obtained at $T = 100$ mK, $B = 9.5$ T and $V_{\rm tip} = 0$, -2 and -4 V, respectively. The top black bar represents 1 μm. (d) R vs $V_{\rm tip}$ with the tip positioned near the saddle point of the QPC. The brown dashed lines indicate the resistance expected for N^*.

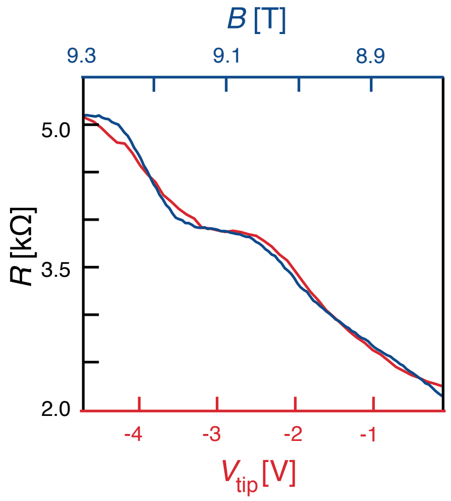

FIG. S 2: QPC resistance vs B (top axis) and $V_{\rm tip}$ (bottom axis) at $T = 4.2$ K.

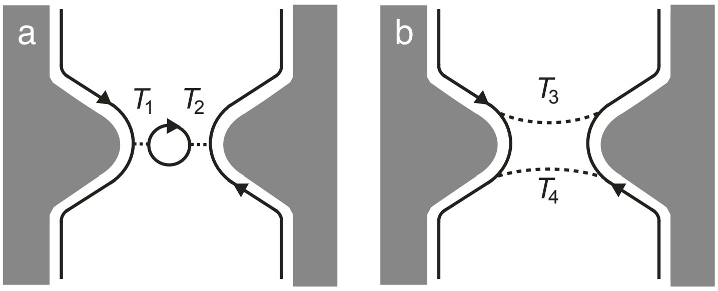

FIG. S 3: (a) Schematic representation of a QHI at the center of the QPC. Tunneling paths (dotted lines) connect opposite ES through the QHI. Γ1 and Γ2 are transmission coefficients of the tunnel barriers between ES and the QHI.. (b) Alternative model for the situation at the QPC: two tunnelling paths (dotted lines) on both sides of the saddle point connect counterpropagating edge states and form a closed loop. Note that in both cases, only one edge state is represented, for the sake of clarity
